# The Emergence of Cutaneous Digital Twins in Dermatology: A Systematic Literature Review

**DOI:** 10.7759/cureus.116858

**Published:** 2026-09-24

**Authors:** Zarafshan Rashid, Javaid Butt

**Affiliations:** 1 Internal Medicine, Birmingham Community Healthcare NHS Foundation Trust, Birmingham, GBR; 2 Additive and Sustainable Manufacturing, Buckinghamshire New University, Buckinghamshire, GBR

**Keywords:** artificial intelligence, cutaneous, dermatology, digital twin, healthcare, personalised medicine, skin

## Abstract

Digital twins (DTs) have made a significant impact on many industrial sectors, including healthcare and biomedicine, where they are being applied to patient-specific digital twinning, personalised medicine, predictive diagnostics, and surgical planning. While the literature is sparse, studies are emerging, with a particular focus on dermatology because many skin-related issues are inherently multimodal, longitudinal, and patient-specific. This has led to the development of cutaneous digital twins (CDTs), which establish an automated, live, and bidirectional relationship between the physical cutaneous system and its digital counterpart. A CDT is therefore a patient-specific and continuously updated virtual model of the skin.

To characterise the current technical landscape of this emerging field, a Preferred Reporting Items for Systematic Reviews and Meta-Analyses (PRISMA)-informed systematic literature review was undertaken. This identified 426 records; following removal of duplicates and non-article records, 248 underwent title and abstract screening, resulting in 16 retained studies. Three domains were identified from the retained studies, with physics-based CDTs for transdermal drug delivery and percutaneous absorption being the most developed, followed by biomechanical CDTs for skin modelling, growth, and tissue expansion, and disease-specific and AI-enabled CDTs being the least mature.

Cross-cutting gaps in CDT development were identified, including limited multimodal integration, underdeveloped longitudinal updating, inconsistent and preclinical validation, weak clinical workflow integration, and the overarching gap between technical enablement and CDT realisation. Overcoming these gaps would require a shift toward workflow-oriented architectures. Manufacturing DT principles can offer a roadmap for this transition by providing an architectural model for connecting simulation, data, feedback, and optimisation within a governed lifecycle. For clinical translation, a human-in-the-loop layer should be introduced within the architecture to ensure that CDT outputs are interpreted appropriately and iteratively refined by clinicians for effective diagnosis.

## Introduction and background

With the emergence of digital twins (DTs), the landscape of engineering and manufacturing has changed drastically due to their vast benefits, including real-time monitoring and decision-making, optimised workflows, predictive maintenance, modelling, and control [1,2]. A DT is a dynamic virtual copy of a physical asset, process, system, or environment that represents and behaves like its real-world counterpart, with continuous and bidirectional real-time data exchange [3,4]. Its functionality is supported by advanced technologies such as the Industrial Internet of Things (IIoT; a network of connected sensors that feeds real-time data into the model) [5], AI [6], physics-based/data-driven simulations for predictions [7,8], and extended reality for visualisation [9]. While the literature and practical implementation of DTs are growing, some misconceptions still linger due to similar approaches to representing physical systems. For example, there are three related concepts of virtual representation that involve developing digital replicas of physical entities, but they differ in their scope, purpose, and linkage to the physical system [10]. These include the DT, digital model, and digital shadow. While a DT involves a fully automated data flow between the virtual and physical entities, the digital model is disconnected from the physical object (requiring manual data exchange), and the digital shadow involves one-way automatic data flow from the physical object to the digital model [11]. These distinctions are important to note to ensure that the appropriate protocol is employed based on the required level of data integration for decision-making [12].

To further enhance the cognitive capabilities of DTs for improved performance and decision-making, research has focused on semantic modelling and ontologies [13], as well as Internet of Things (IoT) data management and knowledge graphs [14]. In practice, this means that the twin can integrate different data types and reason over them, or use a knowledge graph (a structured map of how clinical entities relate to each other) to link a lesion to its history, pathology, and treatment response. This has led to a greater capacity for interpretation and action under changing conditions for DTs. It is important to note here that the cognitive DT is a more sophisticated subset of the DT, with learning, reasoning, and context awareness [15]. With such enhanced capabilities and the dynamic data coupling with physical systems, DTs have gained significant attention in different domains where system complexity, heterogeneity, and the need for predictive insight make conventional modelling approaches insufficient on their own. This translational capability has accelerated adoption across healthcare and biomedical contexts, where DTs are being applied to personalised medicine, predictive diagnostics, and surgical planning [16]. Examples of DTs in medicine include cardiology, neurology, surgery, and oncology, demonstrating their ability to enhance diagnostic accuracy, optimise treatment plans, and improve patient outcomes [17-20]. Like manufacturing DTs, healthcare DTs also utilise IoT and AI and are integrated with advanced methods such as deep phenotyping [21] and digital phenotyping [22] for disease progression simulation and treatment evaluation [23]. The literature highlights the impact of patient-specific DTs but remains diverse in both terminology and technical maturity, ranging from relatively simple digital representations and simulation models to more sophisticated, data-linked systems capable of updating over time and informing clinically relevant decisions [24-26]. Moreover, according to a recent report by MarketsandMarkets, the global DT market is expected to grow from USD 21.14 billion in 2025 to USD 149.81 billion in 2030 at a compound annual growth rate (CAGR) of 47.9%. The healthcare segment is expected to register the highest CAGR, at 52.7% [27], a rate at which the market more than doubles roughly every two years, indicating rapid expansion.

Dermatology is particularly well suited to this development because many skin-related issues are inherently multimodal, visually rich, longitudinal, and patient-specific [17]. Skin is the largest organ and often requires repeated clinical observation, imaging, and therapeutic adjustment, thus making dermatology a plausible setting for digital approaches that aim to combine representation, monitoring, and prediction [28]. Many cutaneous conditions also fluctuate over time and vary substantially between patients. This has led to an increased focus on patient-specific digital twinning rather than one-off static classifications [29]. Within this context, the cutaneous digital twin (CDT; a virtual replica of the skin as a biological system) can be introduced as a dermatology-specific form of digital twinning centred on patient-specific representation, monitoring, and prediction [30]. In practical terms, a CDT would involve a patient-specific digital representation of the skin phenotype, informed by dermatological data and capable of supporting monitoring, simulation, treatment-response modelling, or other clinical activities [28-30]. One prominent early application is the modelling of percutaneous absorption (how drugs and other substances move across the skin layers into the bloodstream), which underpins transdermal drug delivery and recurs throughout this review.

It is also important to note that not every virtual or computational representation of skin should be considered a true CDT. A CDT should be characterised by an automated, live, and bidirectional relationship between the physical cutaneous system and its digital counterpart, which is updated by incoming data and can also support feedback, prediction, and decision-making. At this stage, it is critical to position CDTs within the broader landscape of DTs to ensure a relevant understanding of the underpinning concepts. Therefore, a CDT can be interpreted as a dermatology-specific instantiation of the broader DT paradigm and potentially as a specialised form of cognitive DT when multimodal integration, prediction, and decision support are present. Figure 1 shows the positioning of CDTs within the broader DT landscape. This is an important aspect of this review, as it distinguishes CDTs from generic dermatology-related modelling, image analysis, or AI approaches that do not necessarily achieve the integrative and dynamic features implied by digital twinning [17,28,30]. Specifically, a CDT requires patient-specific representation, bidirectional data coupling with its physical counterpart, dynamic updating, and predictive capacity. Most existing dermatological models meet only one or two of these criteria and therefore fall short of true digital twinning. In short, a true CDT is one that maintains: (1) a patient-specific representation; (2) an automated, live, bidirectional link with the physical skin; (3) updating based on incoming data; and (4) support for feedback, prediction, and decision-making. As an example for clarity and distinction, an AI model that classifies a single photograph of a mole as benign or malignant is not a CDT because it does not maintain an ongoing, updated representation of that patient's skin over time.

**Figure 1:**
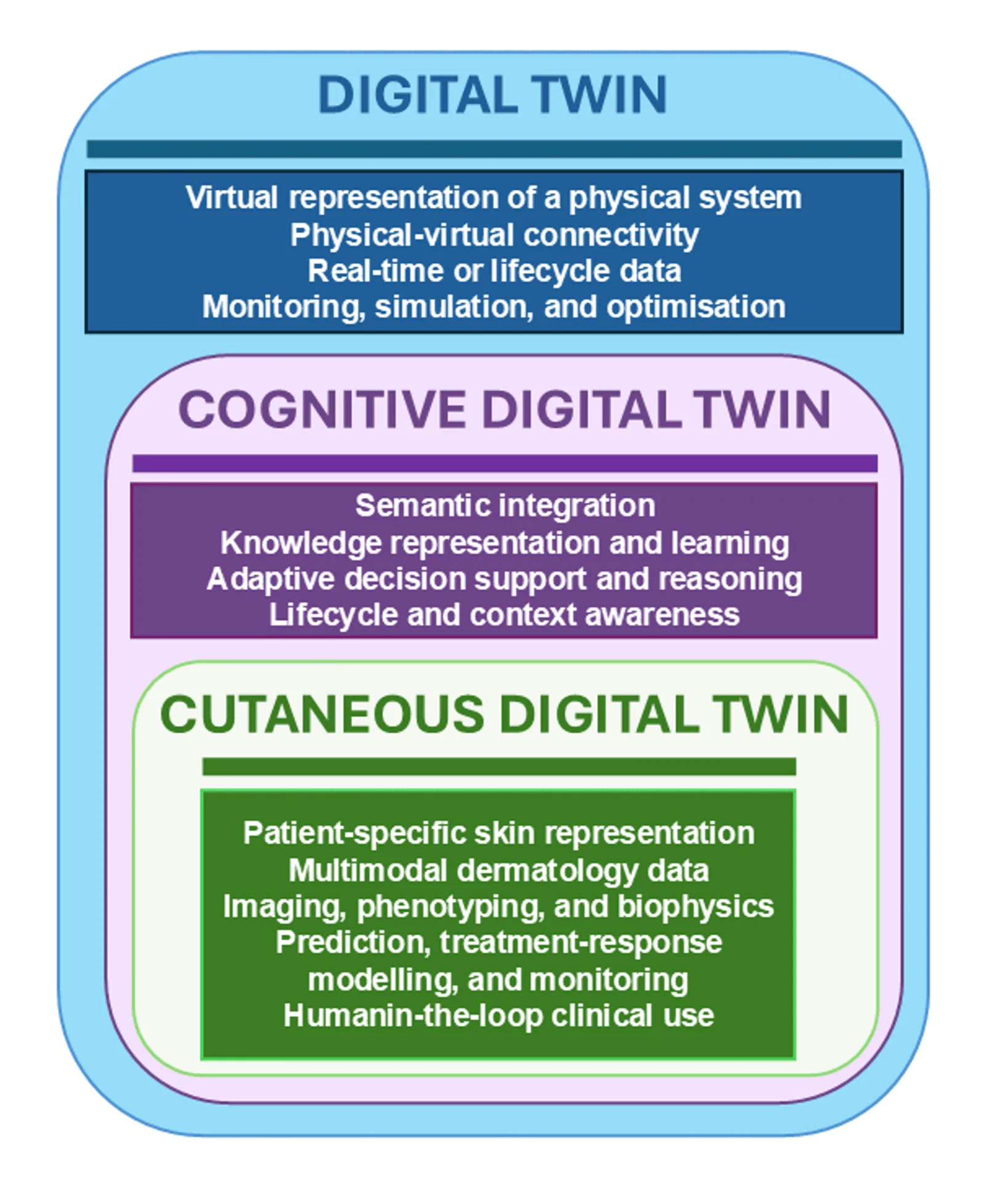
Positioning of cutaneous digital twins within the broader digital twin landscape. Figure was created using Microsoft PowerPoint (Microsoft Corporation, Redmond, WA, USA).

While there is growing interest in healthcare DTs, the literature relevant to CDTs is fragmented and terminologically inconsistent [17,28]. Moreover, it does not reflect the maturity of CDTs but rather describes conceptual ideas [30] or biomechanical modelling (e.g., mechanistic transdermal modelling [31], tissue expansion simulation [32], or disease-specific lesion analytics [33]) supported by AI [34,35], as opposed to a genuine bidirectional DT architecture. At the same time, searches using the term ‘skin’ retrieve a large number of studies that are irrelevant to dermatology and instead focus on engineering, materials, robotics, or other non-cutaneous uses of the term. This review is therefore novel in both scope and method; i.e., it applies a disciplined approach to identify dermatology-specific CDT literature, separating direct CDT contributions, substantively enabling studies, and non-relevant peripheral retrievals. In doing so, it provides a more informative synthesis than prior perspective-based work by showing how CDTs are emerging, the domains in which development is occurring, and the barriers that continue to limit clinical translation.

## Review

Research methodology

For the systematic literature review, the PRISMA 2020 guidelines [36] were adopted, covering the period between 2000 and 24 April 2026, as shown in Figure 2. Identification and evaluation of CDT-relevant publications followed the systematic review procedure outlined in [37], which involves defining research questions, collecting relevant studies from appropriate sources, and critically selecting and evaluating the collected literature. Each primary study was assessed against predetermined inclusion and exclusion criteria. The following subsections describe the five steps involved in the systematic literature review.

**Figure 2:**
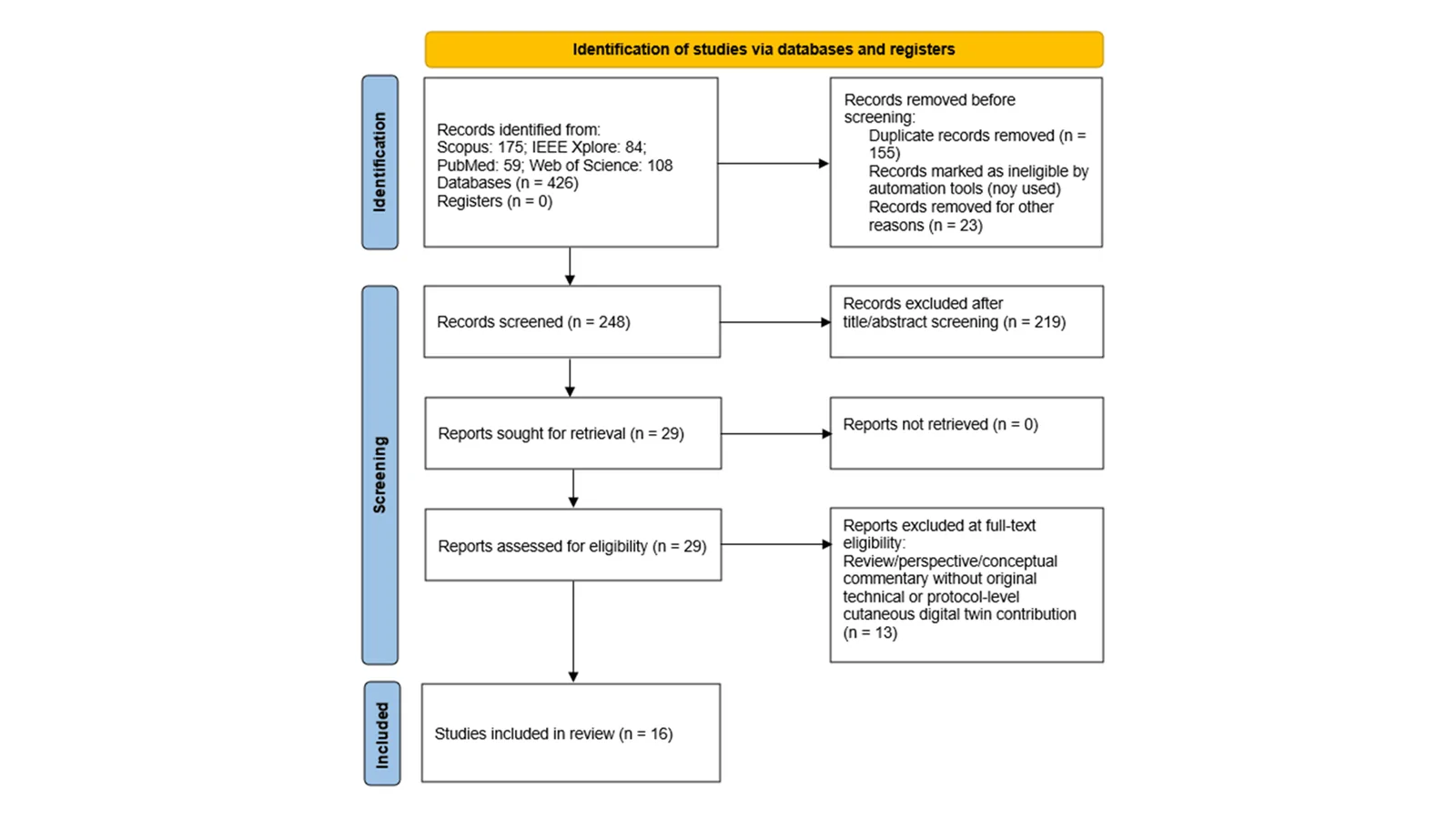
PRISMA 2020 flow diagram for the systematic literature review. PRISMA: Preferred Reporting Items for Systematic Reviews and Meta-Analyses.

Step 1: Potential Research Questions

Four key research questions were formulated for this systematic literature review. They are shown in Table 1 and are answered in the 'Results' section.

**Table 1:** Research questions and their evidence locations. RQ: Research question; CDT: Cutaneous digital twin.

Code	Research question	Evidence
RQ1	What is the current state and temporal evolution of CDT development in dermatology?	Search results and screening outcomes
RQ2	What technical contributions, modelling approaches, and data inputs characterise CDT development in dermatology?	Contributions of the retained studies
RQ3	What substantive domains of CDT development have emerged in dermatology?	Domain-based interpretive synthesis
RQ4	What cross-cutting barriers currently limit the clinical translation and adoption of CDTs?	Cross-cutting patterns and gaps in CDT development

Step 2: Keyword Identification

Appropriate keywords were identified to ensure that a broad range of literature could be captured without making the search too narrow to include relevant emerging terminology. The final search string (using the Boolean operators “AND” and “OR”) used in this analysis was:

(“digital twin*” OR “virtual twin*” OR “cutaneous digital twin” OR “skin digital twin”) AND (dermatolog* OR cutaneous OR skin).

Step 3: Resources for Searching

Databases used to search for relevant literature included Web of Science (https://www.webofknowledge.com), Scopus (https://www.scopus.com), IEEE Xplore (http://ieeexplore.ieee.org), and PubMed (https://pubmed.ncbi.nlm.nih.gov/). These academic databases provide extensive and reliable sources covering the subject matter of this review, offering major collections of peer-reviewed journal articles, books, conference proceedings, and other scholarly publications. The search string was adapted to the syntax of each database. No study registers or other literature sources were searched. The review protocol was not formally registered in any external registry.

Step 4: Inclusion and Exclusion Criteria

Strict inclusion criteria were utilised for this work to ensure the reliability and relevance of the literature for CDT. Studies were retained only when they provided one or more of the following: (1) direct implementation of a cutaneous or skin-related DT, presenting a bidirectional, updating DT of the skin or a cutaneous condition; (2) patient-specific or physics-based cutaneous modelling explicitly within a DT framework; (3) disease- or application-specific CDT architectures, services, or protocol-level translational studies; or (4) strongly enabling cutaneous modelling workflows directly positioned toward DT development. Review papers, broad narrative overviews, and perspective articles without an original CDT-specific framework or technical contribution were excluded from the retained synthesis, although they were still cited where appropriate.

Non-English records were excluded, as were those in which ‘skin’ was mentioned in a non-cutaneous engineering context. Non-dermatological medical DTs without substantive cutaneous relevance and generic dermatology AI/image analysis studies without a strong CDT framing were also excluded. Full-text assessment was also performed to identify the most relevant studies for CDT development in dermatology through strict qualitative synthesis.

Step 5: Data Extraction and Synthesis

Data were extracted from all records for descriptive mapping and strict synthesis. Four elements were recorded from the screened records for completeness, i.e., publication year, title, assigned descriptive category, and screening outcome according to the inclusion/exclusion criteria. The studies were then grouped into categories according to their descriptive relevance to CDT development in dermatology. This descriptive categorisation was used to assess the temporal distribution and relevance of the screened literature. Given the heterogeneity of the evidence base and the lack of comparable quantitative outcomes, meta-analysis was neither feasible nor appropriate.

For the strict synthesis, additional extraction focused on the substantive contribution of each retained study. These fields included CDT relevance, key challenge addressed, main technical or protocol-level development, principal outcome or insight, and major limitation or gap. This second-stage extraction was important to ensure that only studies providing a substantive contribution to CDT development in dermatology were retained, rather than treating all retrieved records as equally informative. The retained records were also synthesised qualitatively through an interpretive domain-based approach, consistent with the methodological heterogeneity of the literature and the lack of sufficiently homogeneous quantitative outcomes to support meta-analysis. Both authors independently screened all records against the eligibility criteria, and disagreements were resolved through discussion until consensus was reached.

Results

Search Results and Screening Outcomes

A total of 426 records were identified from the four databases (Scopus, IEEE Xplore, PubMed, and Web of Science) using the specified keywords. Of the 426 records, 155 were removed as duplicates, and 23 were removed because they represented proceedings/container metadata and other non-article bibliographic entries. For clarity, the remaining 248 records were first descriptively grouped into four categories: explicit cutaneous/dermatology DT, skin-related enabling DT, peripheral health-related DT, and non-cutaneous ‘skin’ retrievals, as shown in Figure 3. These categories allowed for a better understanding of the temporal distribution and scope of the literature. Over time, clear growth can be observed in the incorporation of DTs within the dermatology domain. The number of records increased from 12 in 2020 to 65 in 2025. However, it is important to note that not all of this growth was driven by CDT papers. The retrieved studies were largely dominated by peripheral health-related DT records (n = 175) and non-cutaneous ‘skin’ retrievals (n = 35), with explicit cutaneous/dermatology DT and skin-related enabling DT records amounting to 18 and 20, respectively. It is also worth noting that the explicit cutaneous/dermatology DT literature was not only the smallest category but also began appearing only from 2023 onwards. These are important aspects, as they demonstrate the fragmented nature of the literature and the low maturity of CDT development in dermatology.

**Figure 3:**
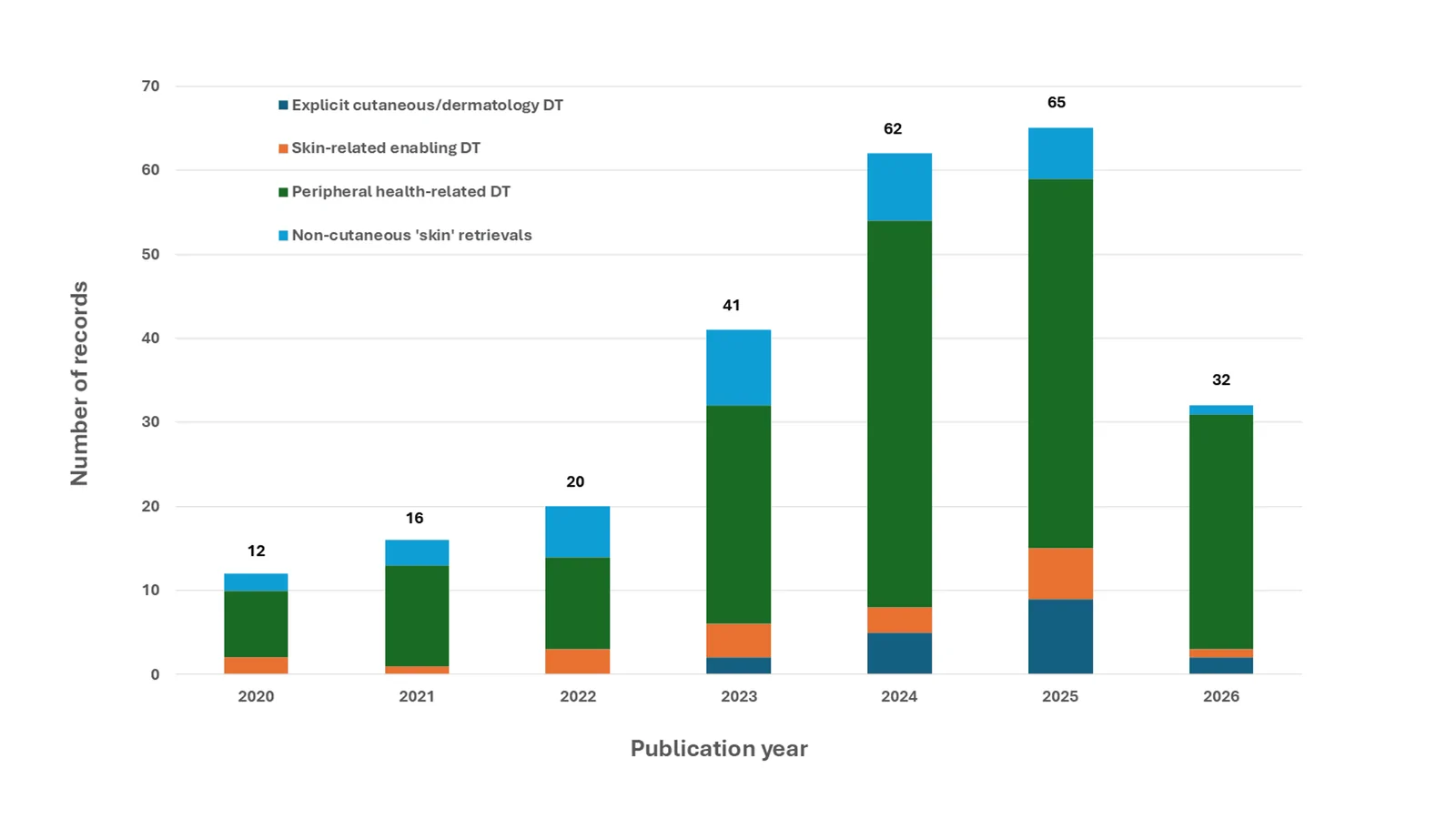
Temporal distribution of retrieved studies by category (n = 248).

After categorisation, title and abstract screening was conducted for the 248 records. Records excluded after title/abstract screening (n = 219) comprised non-English records (n = 9), non-cutaneous ‘skin’ engineering/materials/robotics/environmental records (n = 19), general or non-cutaneous medical DT records (n = 17), and dermatology/skin-related papers without an explicit or substantively enabling CDT contribution (n = 174). Full-text assessment was performed for the remaining 29 records, of which 16 were retained in the strict qualitative synthesis. It is also interesting to note that all 16 records belonged to the explicit cutaneous/dermatology DT and skin-related enabling DT categories, further highlighting the effectiveness of the two-stage synthesis.

Contributions of the Retained Studies

To address research question 2 (RQ2), the contributions of the retained studies to CDT development in dermatology were first summarised, as shown in Table 2. The purpose was to establish the content of the retained evidence base before grouping the studies into broader CDT domains. The retained studies comprised 10 explicit cutaneous/dermatology DT studies and six skin-related enabling DT studies.

**Table 2:** Summary of the retained studies (n = 16). CAD: Computer-aided diagnosis; CDT: Cutaneous digital twin; CNN: Convolutional neural network; D2T-PT: Deep Digital Twin Services for Personalized Treatment; E2E: End-to-end; EMG: Electromyography; FE: Finite element; IMU: Inertial measurement unit; LSTM: Long short-term memory; MPX: Monkeypox; MPXV: Monkeypox virus; MxSLDNet: Monkeypox Skin Lesion Detector Network; PBPK: Physiologically based pharmacokinetics; PD: Pharmacodynamics; PK: Pharmacokinetics; SEB: Squeeze-and-excitation block; XAI: Explainable artificial intelligence; DIC: Digital image correlation.

#	References	Cutaneous DT relevance	Key challenge addressed	Main contribution	Main insight	Limitation/gap
1	Mustafina VS, Sukhov MV (2020) [38]	Early proof-of-concept disease-specific CDT precursor	Utilising psoriasis image analysis as an initial step toward patient digital twinning	CNN-based psoriasis recognition explicitly linked to digital twin ambition	Signals early inflammatory-dermatoses digital twin thinking	Very early-stage; closer to AI precursor than mature CDT
2	Defraeye T et al. (2020) [39]	Physics-based CDT enabling transdermal therapy	Personalising transdermal fentanyl delivery	Validated in silico mechanistic modelling of fentanyl diffusion, storage, and partitioning in the epidermis	Showed that patient- and site-sensitive in silico modelling can support tailored therapy	Limited clinical validation; lacks multimodal integration; narrow application scope
3	Defraeye T et al. (2021) [40]	Inverse mechanistic modelling of CDT workflow for transdermal drug delivery	Fast identification of optimal parameters for transdermal models	Inverse mechanistic modelling for optimal parameter estimation	Improved calibration logic for personalised transdermal twin development	Still model-centric; not a fully operational clinical twin
4	Bahrami F et al. (2022) [41]	Age-sensitive CDT for transdermal therapy	Accounting for age-linked physiological variability	Physics-based in silico simulation framework implementing drug uptake, PK, and PD models for age-sensitive therapy tailoring	Extended transdermal CDT logic to age-specific therapy optimisation	Focused on one therapeutic context; not yet integrated with broader multimodal or prospective clinical workflows
5	Bahrami F et al. (2023) [42]	Individualised virtual patient CDT for transdermal fentanyl therapy	Patient-specific tailoring of chronic-pain transdermal therapy	Explicit individual digital twin architecture linking transdermal uptake with therapy modelling for chronic pain management	Most direct demonstration of individualised transdermal CDT logic within the retained set	Application remains bounded to fentanyl therapy; broader dermatology-wide integration is lacking
6	Atashipour SR, Baqersad J (2023) [43]	Biomechanical CDT of human skin	Non-invasive characterisation of skin mechanics	Vibration-response from the analytical model compared with DIC measurements and FE modelling	Demonstrated non-invasive human-skin digital twin approach	Mechanics-focused and application-specific; limited direct linkage to longitudinal dermatology workflows
7	Bahrami F et al. (2024) [44]	Thermally controlled CDT for transdermal delivery	Reducing intra-individual variability and controlling fentanyl delivery through thermal modulation	Physics-based CDT with an in silico skin model for drug penetration, a PK model, and a PD model; evaluates site effects, ambient temperature, activity level, and patch-temperature control	Showed that thermal control can reduce variability, improve plasma concentration stability, and lower pain intensity	Specialised drug-delivery use case; broader clinical deployment and multimodal integration remain limited
8	Bahrami F et al. (2024) [45]	Physics-based digital patient twin for transdermal opioid switching	Tailoring switch from oral/IV morphine to transdermal fentanyl	Physics-based simulations on a virtually generated patient population; combines patient physiology with opioid therapy overlap duration, fentanyl dose, and PK/PD outcome measures including pain relief and minute ventilation	Showed that age, weight, sex, and combined physiological features materially alter optimal switching strategies and therapy outcomes	Still simulation-based; requires fuller clinical validation
9	Cukic M et al. (2024) [46]	Protocol-level translational testing of CDT logic	Assessing whether rapid CDT-guided fentanyl dosing outperforms standard practice	Pilot study protocol for physiology-based DT logic in a prospective translational design; bridges mechanistic modelling and real-world clinical evaluation	Represented the clearest protocol-level step from simulation toward CDT-guided clinical decision support	Protocol only; modelling done on virtual patients; no completed patient outcome data yet
10	Zhang D et al. (2025) [47]	Substantively enabling physics-based CDT workflow	Making complex percutaneous absorption modelling E2E and accessible to allow non-modelling experts to simulate transdermal permeation	E2E in silico workflow for percutaneous absorption simulation, integrating diffusion-partition properties and PBPK dermal absorption modelling	Showed that complex formulation and exposure effects on percutaneous absorption can be simulated accurately in a CDT-oriented workflow	Workflow-enabling rather than fully patient-specific CDT; limited evidence of integrated clinical deployment
11	Sampath P et al. (2025) [48]	Disease-specific and AI-enabled CDT application in skin oncology	Accurate and interpretable skin cancer classification with lower computational burden within CDT framing	CAD-based explainable digital twin framework using early activation feature extraction, machine-learning classification, and XAI for skin cancer analysis	Presented a CDT-informed framework to simulate and analyse the diagnostic process virtually, enabling real-time feedback and performance monitoring	Closer to an AI-enabled diagnostic twin framework than to a fully coupled longitudinal CDT system
12	Gabriel CIO et al. (2025) [49]	Disease-specific CDT model for infectious lesions	Personalised MPX treatment and monitoring in real time to contain the spread of the virus	Introduced the D2T-PT model, combining transfer learning and DT to improve the accuracy of MPX detection and real-time monitoring, supported by the SEB attention mechanism	Service-oriented digital-twin support for personalised MPX treatment using CNN and advanced adaptive NASNetMobile to achieve highly accurate results	Early-stage; limited validation and narrow disease scope
13	Laudo J et al. (2025) [50]	Biomechanical CDT for tissue expansion	Calibrating digital twins for human skin growth	Development and calibration of skin-growth digital twins using finite element models from 3D photos	Strong mechanics-based CDT contribution with clear clinical relevance	Application remains concentrated in tissue expansion
14	Sharma V et al. (2025) [51]	AI-enabled disease-specific CDT application	MPX detection with DT-based lesion analytics requiring less storage	Introduced an automated MxSLDNet framework for MPX lesion detection and benchmarking against pre-trained models	Rapid extension of the CDT workflow into infectious lesion analytics, helping with diagnostic and treatment planning	More predictive and AI-enabled rather than fully bidirectional or continuously updating CDT
15	Laudo J et al. (2025) [52]	Substantively enabling biomechanical CDT study	Predicting deformation during tissue expansion and reconstruction	3D photography-informed FE pipeline with uncertainty quantification, Bayesian updating, and Gaussian-process surrogate modelling	Strengthened patient-specific biomechanical CDT development	Limited breadth and longitudinal workflow integration; broader dermatological generalisation is limited
16	Charafeddine J et al. (2026) [35]	Hybrid AI-biomechanics CDT contribution	Real-time prediction and mitigation of socket-related skin damage	Multimodal DT combining EMG, pressure, temperature, IMU data, viscoelastic tissue modelling, CNN–LSTM risk prediction, and rule-based adaptation	Extended CDT logic toward damage prediction in applied biomechanical settings with five transtibial users	Small pilot; simplified tissue model; limited long-term and clinical validation

Domain-based Interpretive Synthesis

A strict qualitative synthesis was performed on the retained studies to identify substantive CDT development domains in dermatology. Three domains were derived inductively. Both authors independently grouped the 16 retained studies according to modelling approach, data inputs, and clinical application, and then reconciled their groupings through discussion to reach the final structure. These domains sit at different maturity levels, but they should not be considered fixed or mutually exclusive. Addressing research question 3 (RQ3), they represent the technical landscape of CDT development in dermatology and are illustrated in Figure 4, together with their interrelationships and shared inter-domain concepts.

**Figure 4:**
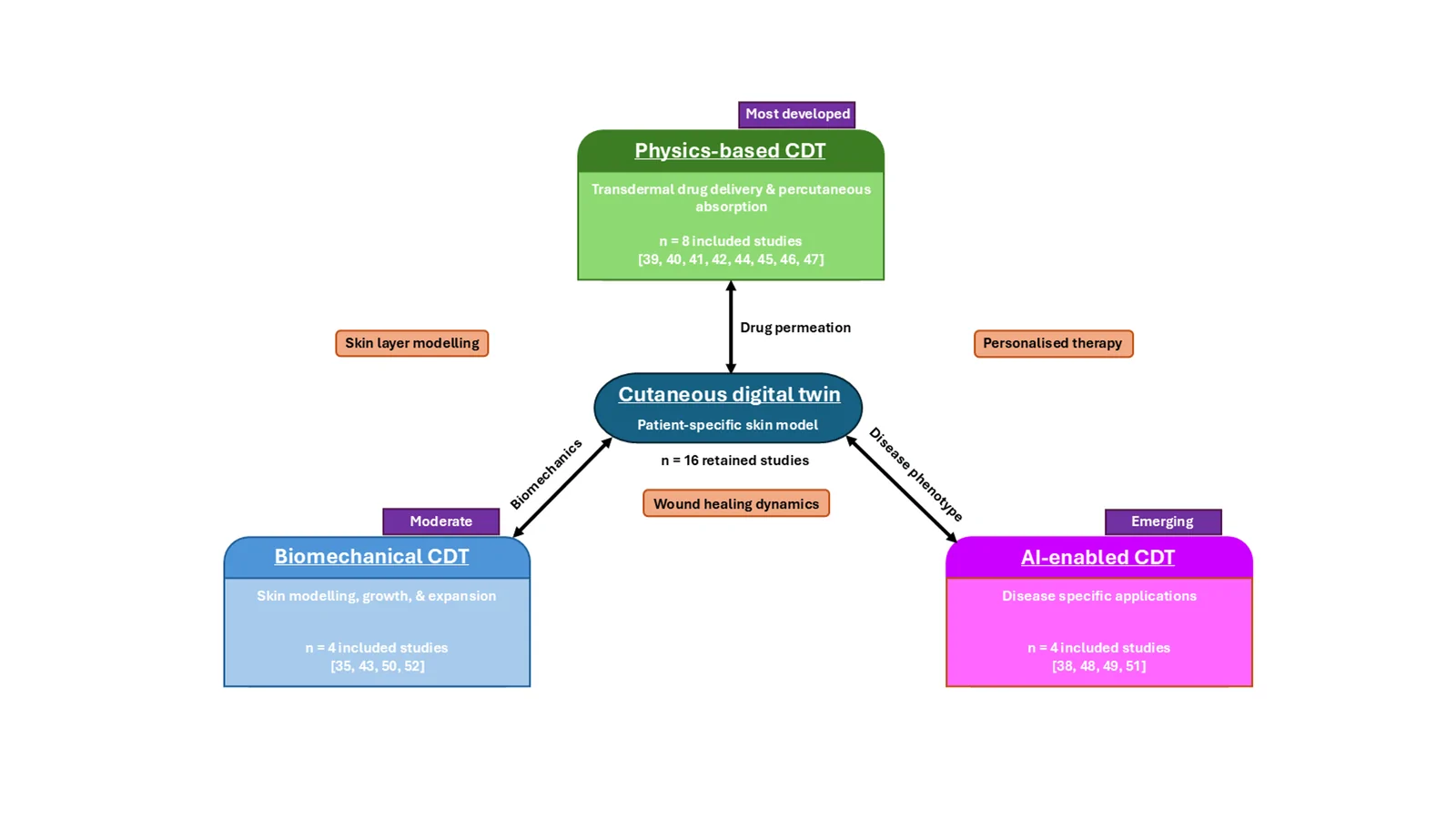
Domains of cutaneous digital twin development in dermatology. Figure was created using Microsoft PowerPoint (Microsoft Corporation, Redmond, WA, USA).

The first and most developed domain comprises physics-based CDT studies for transdermal drug delivery and percutaneous absorption. It encompasses validated in silico mechanistic simulation, inverse modelling, age-sensitive and patient-specific fentanyl delivery, thermally controlled therapy, protocol-level translational testing, and end-to-end percutaneous absorption workflows [39-42,44-47]. While this domain represents the most mature CDT trajectory, it remains concentrated in a narrow therapeutic space, with limited real-world clinical validation and weak multimodal integration beyond physiological and drug-delivery variables.

The second domain, biomechanical CDTs for skin modelling, growth, and tissue expansion, includes studies that replicate the mechanical behaviour of skin as a viscoelastic and multilayered material. These studies use approaches such as vibration-response modelling, patient-specific finite element analysis, 3D photography, uncertainty quantification, viscoelastic tissue modelling, and hybrid AI-biomechanics architectures [35,43,50,52]. These studies are highly relevant to CDT development because they connect patient-specific anatomy or sensor data with predictive mechanics, but they have not yet expanded to the wider range of dermatological diseases and clinical pathways.

The third domain, AI-enabled CDTs for disease-specific applications, is the most heterogeneous of the three and represents an emerging stage of CDT development. It encompasses explainable AI for skin cancer analysis [48], convolutional neural network (CNN)-based psoriasis recognition from images [38], and deep-learning DT frameworks for infectious diseases [49,51]. This domain is gaining attention but is also the least mature, with uneven CDT depth. The studies are closer to AI-enabled predictions than to fully coupled and dynamically updating DTs.

Cross-Cutting Patterns and Gaps in CDT Development

The strict synthesis of the retained studies revealed several cross-cutting gaps irrespective of domain, directly addressing research question 4 (RQ4). First, multimodal integration remains limited. While the CDT concept implies the integration of different elements (sensor data, mechanistic modelling, AI, clinical examination, imaging, pathology, and physiology) [16,24,25], most retained studies relied on a narrow set of inputs. Transdermal studies were strongest mechanistically but concentrated on physicochemical and physiological variables; biomechanical studies incorporated patient-specific image/sensor data without linking them to broader clinical/dermatological records; and AI-enabled studies prioritised single-outcome classification over integrated patient modelling.

Second, longitudinal updating remains underdeveloped. An authentic CDT should evolve as the patient, disease state, treatment response, or skin condition changes over time [17]. However, most retained studies focused on single-task simulation or one-off prediction workflows rather than continuously updated systems. Third, outcome-level validation is inconsistent and largely preclinical [1]. While some studies were validated against experimental, in vitro, or pilot data, no retained study demonstrated that deploying a CDT within a clinical workflow led to measurably improved dermatological decisions, treatment responses, or patient outcomes. Fourth, clinical workflow integration is weak. While CDT outputs are framed as informing clinical decisions, the retained studies stop short of demonstrating how those outputs would be reviewed, confirmed, acted upon, and re-entered through verified follow-up data. This remains critical because clinically meaningful CDTs require interpretability, accountability, governance, human-in-the-loop oversight, and iterative updating based on validated outcomes [30].

Fifth, and underlying all of the above, a gap exists between substantively enabling technologies and CDT realisation. The retained evidence base is component-rich but architecture-poor, showing a variety of validated modelling capabilities without demonstrating how those capabilities translate into a patient-specific, continuously updating, clinically governed system. Component technologies are maturing faster than the integrative architectures needed to deploy them as clinical CDTs [11]. To close this gap, workflow-level thinking is required to connect technical capability with clinical context, feedback, and governance. In practice, this means specifying the flow of data, model updates, and coordinated clinical decisions that are validated and audited within a single governed pipeline. Defining such reference architectures, rather than further optimising individual components, is likely to be the rate-limiting step for clinical CDT translation.

Discussion

Principal Findings

This systematic review highlights that CDT development in dermatology is emerging from a small but technically diverse evidence base. The retained studies demonstrate strong technical advancements but lack a mature clinical CDT ecosystem. Transdermal delivery and percutaneous absorption show the most progress because the target problems are bounded and well suited to physics-based CDTs. Biomechanical CDT studies show strong technical promise but remain concentrated in specialist contexts. Disease-specific and AI-enabled applications are expanding, but their CDT status is the least mature, as many studies are closer to diagnostic AI than to integrated, dynamically updated twins. Overall, the findings suggest that dermatology has made strong advances in modelling techniques but lacks a coherent workflow for transforming them into clinically validated CDT systems.

From Models to Workflow-Oriented CDTs

The gaps identified in the 'Cross-Cutting Patterns and Gaps in CDT Development' section suggest that the next phase of CDT development should focus on system-level design by integrating multimodal data, validated modelling, human-in-the-loop interpretation, iterative feedback, and clinical governance within a coherent dermatology workflow [1,11,16,17,24,25,30]. For this, it is crucial to consider other technical fields where DTs have matured. One good example is manufacturing, where DTs have evolved from simulation models to lifecycle systems that connect physical entities, virtual models, real-time data, and feedback [53-58]. In manufacturing, a DT is not defined by a single simulation but by the continuity of its connection to the physical asset, the bidirectional data flow between them, and the capacity to support ongoing monitoring, prediction, and decision-making.

The retained studies show that dermatology has sophisticated modelling tools across all three identified domains. There is, however, a lack of a coherent workflow for connecting those tools to clinical data and interpretation, feedback, and longitudinal updating. An authentic workflow-oriented CDT for dermatology should focus on multimodal data acquisition (e.g., clinical examination, imaging, dermoscopy, pathology, physiology, and longitudinal follow-up), feeding into a patient-specific virtual representation capable of simulating absorption, deformation, lesion behaviour, or treatment response. The output would then pass through a human-in-the-loop clinical layer, where dermatologists confirm interpretations, contextualise model outputs against pathology, and feed verified clinical information back into the model. This creates a learning loop that distinguishes a clinical CDT from a technically sophisticated but disconnected simulation. This workflow perspective directly addresses the five gaps identified in the 'Cross-Cutting Patterns and Gaps in CDT Development' section and is specifically responsive to the gap between technical enablement and CDT realisation.

Within this context, the proposed workflow maps directly onto the cognitive DT concept highlighted in the 'Introduction' section. As established in the Introduction, cognitive DTs extend conventional DTs by incorporating semantic integration, contextual reasoning, and adaptive decision support [15]. The workflow described here represents a progression toward this cognitive tier by adding a multimodal clinical layer with human-in-the-loop interpretation and builds toward a system that learns, updates, and adapts its representation through verified clinical feedback. The retained studies represent the early modelling layers of this progression, and the cognitive CDT represents its mature clinical endpoint. This framing can bridge the gap between the technical promise demonstrated in the retained studies and their current distance from clinical deployment.

It should be noted that manufacturing DT principles should not be imported directly into dermatology because patients are not industrial assets. In manufacturing, DTs mirror a physical asset throughout its lifecycle, continuously ingesting sensor data to monitor performance, predict failures, and optimise operation via closed-loop feedback. It is these architectural principles, rather than the industrial context itself, that translate usefully to dermatology. In this context, the inherent medical complexities related to ethics, equity, governance, and clinical uncertainty require attention. That said, the architectural principles of connectivity, lifecycle updating, service orientation, and feedback-driven validation offer a conceptual roadmap that dermatology currently lacks, and the cognitive DT framework provides the clinical and epistemic ambition that should define its next developmental phase.

Implications for Clinical Translation

The retained studies clearly highlighted limited clinical translation and cross-cutting gaps. Therefore, it is imperative that CDT outputs are interpretable, clinically actionable, prospectively validated, and safe within dermatology workflows. Clinical translation will require a clearer framework for multimodal integration, modelling, validation, uncertainty estimates, and decision pathways. In this context, the human-in-the-loop layer becomes a core requirement. It can provide oversight, diagnostic confirmation, feedback, and accountability. This becomes particularly critical because the use of AI can present disparities across skin tones and uncommon diseases [59], making equitable, phototype-stratified validation an essential requirement for wider CDT implementation. Realising these requirements will depend on regulatory validation standards and multidisciplinary collaboration among clinicians, medical professionals, and data scientists from the design stage onward. Embedding equity and oversight retrospectively is unlikely to succeed. Therefore, these requirements must be specified as core design constraints rather than downstream checks.

Strengths and Limitations

The major strength of this systematic review is its strict distinction among broad retrieval, descriptive mapping, and retained synthesis. This was necessary, as previous studies have reported extremely limited use of DTs in dermatology [17,28]. Moreover, searches for ‘digital twin’ and ‘skin’ retrieve many peripheral health records and non-cutaneous uses of the word ‘skin’. This review avoids overstating the maturity of the field by separating direct and substantively enabling CDT contributions from adjacent or misleadingly retrieved work.

The main limitation is that the retained evidence base remains small and sensitive to definitional boundaries. On the one hand, some substantively enabling studies do not explicitly claim to be CDTs but are building toward them. On the other hand, some explicit CDT papers are closer to AI-enabled analysis and predictions than to fully developed twins. This boundary problem is unavoidable in an emerging field because such fields often mature through bounded technical advances. However, it was managed through transparent screening and synthesis logic. It is also important to note that the retained studies were methodologically heterogeneous and did not share a common design. Therefore, no single validated risk-of-bias or quality-appraisal instrument could be applied uniformly across them. Study quality was instead governed by the pre-specified inclusion and exclusion criteria, which functioned as the screening safeguard.

Future Directions

Future CDT research should prioritise five areas. First, the field needs greater terminological clarity so that CDT studies can be retrieved and compared more consistently. A reporting standard distinguishing static models, digital shadows, and true bidirectional twins would be beneficial [15,54]. This will ensure traceability and accountability at different levels. Second, richer multimodal datasets are needed, particularly those linking imaging, pathology, physiology, treatment response, and longitudinal monitoring. The benefit would be better monitoring and control practices for patient care. Third, validation strategies should move beyond isolated technical performance metrics toward clinically meaningful assessment. This is critical to ensure the effective and trustworthy application of appropriate strategies. Fourth, a human-in-the-loop workflow layer should be introduced for clinical confirmation and iterative twin updating to ensure compliance and consistency of operation. Fifth, implementation science, governance, interoperability, and equity must be integrated much earlier into framework design. These five priorities are interdependent rather than sequential. Terminological clarity, richer data, robust validation, human oversight, and early governance must advance together to move CDTs from technical proof of concept to clinically trustworthy systems.

## Conclusions

Cutaneous digital twins in dermatology are emerging from a technically diverse but still developing and heterogeneous evidence base. Current studies are concentrated around physics-based percutaneous modelling, biomechanical skin modelling, and early AI-enabled disease applications. The major challenge is the lack of integrated, clinically validated, human-in-the-loop workflows that connect data, simulation, clinical interpretation, feedback, and updating. Manufacturing DTs provide a useful roadmap for CDT development by highlighting lifecycle connectivity, validation, and optimisation. A clinically meaningful CDT should be more than a single simulation model. It should represent a patient-specific, continuously updated dermatology workflow that connects simulation, clinical interpretation, and verified feedback within a governed system that evolves with the patient. Realising this vision could shift dermatology toward personalised and anticipatory care, in which treatment is simulated and optimised before it reaches the patient. Bridging the current gap between component maturity and integrated deployment is the essential next step in genuine CDT development.
